# New therapeutic protocol for improvement of endometrial receptivity (PRIMER) for patients with recurrent implantation failure (RIF) – A pilot study

**DOI:** 10.5935/1518-0557.20190035

**Published:** 2019

**Authors:** Felipe Dieamant, Laura D. Vagnini, Claudia G. Petersen, Ana L. Mauri, Adriana Renzi, Bruna Petersen, Mariana C. Mattila, Andreia Nicoletti, Joao Batista A. Oliveira, Ricardo Baruffi, Jose G. Franco Jr.

**Affiliations:** 1 Center for Human Reproduction Prof Franco Jr. Ribeirão Preto, Brazil; 2 Paulista Center for Diagnosis Research and Training. Ribeirão Preto, Brazil

**Keywords:** recurrent implantation failure, endometrial receptivity, therapeutic protocol, granulocyte colony-stimulation factor, platelet-rich plasma, PRIMER

## Abstract

**Objective:**

To evaluate whether or not one should use a new Protocol for Endometrial Receptivity Improvement (PRIMER) based on platelet-rich plasma (PRP) and granulocyte colony-stimulation factor (G-CSF) to enhance ongoing pregnancy rates in patients with recurrent implantation failure (RIF).

**Methods:**

Women undergoing IVF/ICSI were prospectively divided into two groups: - PRIMER/RIF group (n:33): patients with RIF (defined as ≥2embryo transfers (ETs) and at least 5 morphologically good embryos transferred) in which intrauterine PRP injection and subcutaneous G-CSF-injection were performed. - Control group (n:33): patients in their first IVF/ICSI attempt/cycle (without PRP or G-CSF injection). The PRP was prepared using autologous fresh-whole blood processed to increase platelet-concentration in 2 to 4 fold. All patients undergoing the PRP-treatment received 0.7ml of it through intrauterine-injection 48 hours before the ET. G-CSF (300mg/0.5ml) started simultaneously to PRP and was administered subcutaneously every week.

**Results:**

Regarding implantation, clinical pregnancy and miscarriage rates, we found no statistically significant difference (18.2% *versus* 17.6%, *p*=0.90; 36.4% versus 30.3%, *p*=0.61 and 25.0% *versus* 9.0%, *p*=0.43, respectively). The use of PRIMER enabled RIF patients (previous ET µ: 4.0±1.5) to reach similar ongoing pregnancy and live birth rates like those patients who had their first IVF/ICSI cycle attempt (27.3% *versus* 27.3%, *p*=0.99).

**Conclusions:**

Our results showed, for the first time, evidence that this therapeutic protocol (PRIMER) could be used as a feasible treatment based on biological rationale for patients with RIF, considering its promising outcomes, it is a simple procedure and not associated with patient complications.

## INTRODUCTION

The success of a treatment in assisted reproductive technique (ART) cycles depends on the perfect synchrony between embryonic development and endometrial receptivity. The implantation process requires endometrial growth and differentiation of endometrial stromal cells. Human endometrium contains growth factors, receptors for growth factors, cytokines, and other key factors for correct embryonic and endometrial development. Multiple embryos fail to implant, and a relevant percentage of IVF/ICSI treatment failures are due to endometrial receptivity disorders (Blois *et al*., 2011; Farimani *et al*., 2017).

Recurrent implantation failure (RIF) is a common concern among researchers, physicians and patients. Couples desperately require further diagnostic investigations and/or the use of adjunctive/alternative treatments in order to improve their pregnancy likelihoods. In fact, RIF has been the target of a wide scientific debate and several approaches have been developed in order to solve this reproduction issue (Bos-Mikich *et al*., 2019). Endometrial injury, intrauterine human chorionic gonadotrophin (hCG), endometrial receptivity array (ERA), Preimplantation genetic testing for aneuploidy (PGT-A), “Omics” tools (Genomics, Transcriptomics, Proteomics, Metabolomics), and other techniques have been used in patients with RIF, but the results found still require further analyses for routine use in IVF/ICSI cycles (Somigliana *et al*., 2018; Nardo *et al*., 2015; Spencer *et al.*, 2016; Macklon, 2017; Hviid & Macklon, 2017. The granulocyte colony-stimulating factor (G-CSF) and Platelet-rich Plasma (PRP) are among these new therapeutic approaches for RIF.

PRP is prepared from fresh whole blood and contains several growth factors and cytokines, including vascular endothelial growth factor (VEGF), transforming growth factor (TGF), platelet-derived growth factor (PDGF) and epidermal growth factor (EGF). Therefore, it may help regulate endometrial cell migration, attachment, proliferation, differentiation, and neoangiogenesis, resulting in beneficial effects on endometrial receptivity. The use of Platelet-rich Plasma (PRP) in human reproduction is growing, and it could be a new tool to improve clinical outcomes in patients undergoing ART procedures (Zeyneloglu & Onalan, 2014; Chang *et al*., 2015; Magdi *et al*., 2017). PRP has been used in assisted reproduction, especially in patients with thin endometria (Dhillon *et al*., 2012; Lee *et al*., 2013). 

G-CSF is a hematopoietic lineage-specific cytokine, associated with cell proliferation and differentiation, produced by reproductive tissue cells. This cytokine promotes endometrial immunomodulation and optimizes the interaction between the embryo and the endometrium. Studies have shown that its use is associated with higher pregnancy rates and lower miscarriage rates (Lédée *et al*., 2013; Rahmati *et al*., 2015; Arefi *et al*., 2018).

Considering the beneficial effects of PRP and G-CSF on endometrial receptivity, in addition to the fact that failures in IVF/ICSI cycles are related to endometrial disorders, we conducted a pilot study to evaluated if a new therapeutic Protocol for Improvement of Endometrial Receptivity (PRIMER) based on the use of PRP and G-CSF together can improve ART outcomes in patients with RIF.

## MATERIAL AND METHODS

### Population

A total of 66 patients enrolled in the IVF/ICSI program at the Prof Franco Jr. Center for Human Reproduction, from February 2017 to October 2017, and they were prospectively included in this study. We obtained a complete medical/surgical history from all patients, and total screening was performed to exclude RIF causes (a normal karyotype for her and her partner, and no evidence of uterine defects, ultrasonographic evidence of hydrosalpinx, infections, endocrine problems, coagulation defects, thrombophilia and autoimmune defects). We considered only one fresh embryo transfer cycle. We excluded transfer cycles of frozen embryos.

The women were divided into two groups:

-PRIMER/RIF group: patients with RIF in which intrauterine PRP injection and subcutaneous G-CSF injection were performed. RIF was defined as ≥2embryo transfers (ET), and at least 5 good-morphological embryos were transferred.

-Control group: patients in their first IVF/ICSI cycle attempt (without PRP or G-CSF).

The two groups were matched using the Key Performance Indicators Score (Franco Jr *et al*., 2017) based on female-age, anti-Müllerian hormone (AMH) levels, number of metaphase-II oocytes, fertilization rates, and morphological quality of embryos-transferred.

### Ovarian stimulation protocol

All patients included in the study were submitted to the same ovarian stimulation protocol: the long gonadotropin releasing hormone (GnRH) agonist protocol (GnRH-a) as previously described (Oliveira *et al*., 2012). The starting FSH dose was based on the patient's age, anti-Müllerian hormone level and antral follicle count (Ovarian Response Prediction Index calculation) (Oliveira *et al*., 2012).

To induce final oocyte maturation we administered 250µg of recombinant human chorionic gonadotropin (r-hCG; Ovidrel; Serono, Brazil) subcutaneously when at least two follicles reached a mean diameter of ≥17mm. GnRH-a was administered until the day of the r-hCG injection. Oocytes were retrieved by a transvaginal aspiration under ultrasound guidance, 34-36 hours following the r-hCG injection.

### ART procedures

All metaphase II oocytes received ICSI, which was carried out as previously described (Mauri *et al*., 2010; Oliveira *et al*., 2011). The oocytes were examined after 17-20h to assess fertilization; zygotes with two distinct equal-sized pronuclei were considered normal.

The embryos were routinely transferred after 96h in culture, and supernumerary embryos were cryopreserved. The embryos were then transferred with a Frydman catheter (Frydman^®^ Classic Catheter 4.5 CCD Laboratoire C.C.D; Paris, France) guided by abdominal ultrasound, using a 3.5-MHz convex transducer (Aloka SSD-1100; Aloka Co. Ltd, Tokyo, Japan). A single physician performed all embryo transfers, and only easy transfers (i.e. the catheter passed smoothly through the cervix without the need for uterine fixation clamps) with clear visualization of the catheter tip upon ultrasound were considered. All the patients received luteal phase supplementation with vaginal natural progesterone (Utrogestan^®^; Besins Healthcare, São Paulo, Brazil).

### Platelet-rich Plasma

PRP was prepared using autologous fresh whole blood and a double-spin method. In brief, blood was obtained by venipuncture in syringes containing acid citrate dextrose (ACD-A) solution (1:4 vol/vol). The citrated blood was centrifuged for 12 min at 1400 rpm. Subsequently, the supernatant plasma was taken up using a micropipette and transferred into another sterile tube for centrifugation. A second round of centrifugation was performed for 7 min at 3200 rpm. Platelets pellet formed at the bottom of the tube. The supernatant was removed in another sterile tube and the platelets were suspended in a minimum quantity of plasma by gently shaking the tube. The process increases platelet-concentration in 2 to 4 fold.

All patients undergoing the PRP-treatment (study group) received 0.7ml of it through intrauterine injection, using a soft catheter, 48 hours before the ET.

### Granulocyte colony-stimulating factor

On the same day of the PRP injection, we injected G-CSF (Filgrastim^®^, Biosintetica/Achê, 300µg/0.5ml) subcutaneously, and repeated it weekly. If pregnancy occurs, G-CSF is maintained until the 12^th^ gestation week.

### Endpoints

The primary endpoints were ongoing pregnancy and live birth rates. The secondary endpoints included implantation, clinical pregnancy and miscarriage rates.

### Statistical analysis

Data management and univariate analysis were carried out using the StatsDirect statistical software version 2.7.9 software (Cheshire, UK). The following parameters were evaluated: the woman's age, infertility etiology, number of oocytes retrieved, number of oocytes in metaphase II retrieved fertilization rate, the number of embryos transferred, embryo implantation rates, miscarriage rates, ongoing pregnancy rates and live birth rates. We used the nonparametric Mann-Whitney test to compare the means of continuous variables, when the continuous variables were not normally distributed, and the Student's t-test was used if the continuous variables were normally distributed. The results are expressed as the arithmetic means ± standard deviation (SD). For categorical variables, we used the Fisher's exact test to check between group associations, and the results were expressed as percentages. A *p* value < 0.05 was considered statistically significant.

## RESULTS

We found an equal distribution (*p*>0.05) of the general and cycle's characteristics for PRIMER/RIF (PRP injection and subcutaneous G-CSF injection) and Control (without PRP or G-CSF) groups. Table 1 summarizes the data.

**Table 1:** General and cycles’ characteristics

	PRIMER	Control	*p*
n	33	33	
Previous ET (n)	4.0±1.5	----	
Previous embryos transferred (n)	9.1±2.1	----	
Female age (y)	37.8±3.8	37.8±3.9	0.94
Male age (y)	41.8±4.8	39.1±6.2	0.10
AMH (ng/dl)	2.4±2.7	2.7+4.7	0.43
BMI (Kg/m^2^)	24.4±3.3	24.8±3.9	0.23
Etiology of infertility -Male factor (%) -Tubal peritoneal (%) -Idiopathic (%) -Endometriosis (%)	42.4%(14/33) 12.2%(4/33) 24.2%(8/33) 21.2%(7/33)	36.4%(12/33) 9.1%(3/33) 33.3%(11/33) 21.2%(7/33)	0.62
FSH total dose (IU)	3245.9±1357.7	2866.4±1125.8	0.23
LH total dose (IU)	1398.3±151.2	1084.8±160.4	0.30
Oocytes retrieved (n)	7.8±4.4	6.4±3.6	0.14
MII oocytes (n)	6.1±3.5	5.3±3.1	0.35
Fertilization rate %	60.6±23.2	68.2±25.5	0.14
embryos transferred (n)	2.3±0.9	2.2±0.9	0.53
Good quality embryo transferred (%)	54.5%(18/33)	60.6%(20/33)	0.62

There were no significant differences between the PRIMER and Control groups regarding implantation, pregnancy, spontaneous miscarriage, ongoing pregnancy or live birth rates *p*>0.05). Table 2 shows the main results.

**Table 2:** Main results

	PRIMER	Control	*p*
Implantation rate (%)	18.2% (14/77)	17.6% (12/68)	0.90
Clinical pregnancy rate (%)	36.4% (12/33)	30.3% (10/33)	0.61
Miscarriage rate (%)	25.0% (3/12)	9.0% (1/10)	0.43
Ongoing pregnancy rate (%)	27.3% (9/33)	27.3% (9/33)	0.99
Live birth rate (%)	27.3% (9/33)	27.3% (9/33)	0.99

## DISCUSSION

Our results indicated that the PRIMER enabled patients with RIF (mean number of previous ET of 4.0±1.5) to reach similar ongoing pregnancy and live birth rates to those patients who had their first IVF/ICSI cycle attempt, demonstrating possible beneficial effects on endometrial receptivity. Unfortunately, to the best of our knowledge, the present study is the first to analyze the efficacy of intrauterine PRP associated with subcutaneous G-CSF for RIF patients, and thus cannot be compared with other results. However, PRP and G-CSF have been employed singly in several medical fields, including human reproduction, with promising outcomes, corroborating our findings.

Previous trials have shown the beneficial effects of PRP use on tissues, promoting cell-differentiation, proliferation, growth, and neoangiogenesis (Aghajanova *et al*., 2016). These effects are probably because it contains and/or stimulates several growth factors such as TGF-β, PDGF, IGF, VEGF, EGF and FGF-2 and bioactive-cytokines, which stimulate the inflammatory cascade and the healing process (Le *et al*., 2019). These PRP positive effects could improve reproductive outcomes in patients with impaired endometrial receptivity. However, few trials have evaluated the effectiveness of autologous PRP in patients with endometrium disorders (Chang *et al*., 2018; Tandulwadkar *et al*., 2017; Zadehmodarres *et al*., 2017; Eftekhar *et al.,* 2018; Molina *et al*., 2018; Mehrafza *et al*., 2019).

Regarding the use of G-CSF, studies have shown that its use could improve endometrial thickness and pregnancy rates in patients presenting suboptimal endometria (Jain *et al*., 2018). G-CSF is a glycoprotein, secreted from endothelial cells, macrophages and some other immune system cells. Recently, it has been used in assisted reproduction for patients presenting thin endometrium and/or recurrent pregnancy loss. Some studies have demonstrated that the G-CSF use by transvaginal endometrial perfusion or subcutaneous way could improve ART outcomes, such as implantation and miscarriage rates (Würfel *et al*., 2010; Zafardoust *et al*., 2017; Zhang *et al*., 2018). However, the results in the general population undergoing ART are questionable (Barad *et al*., 2014; Jain *et al*., 2018).

Considering PRP is obtained through fresh-whole blood peripheral vein, its intrauterine-injection is relatively safe, with low-risk of disease-transmission, immune reactions, and deleterious effects to the patient (Welte, 2014). On the other hand, besides G-CSF beneficial effects on reproductive tissues, it is considered safe within the dose and route of administration usually employed (Jang *et al*., 2017). Therefore, the combined use of subcutaneous G-CSF and intrauterine PRP - PRIMER - should be considered as low risk for IVF/ICSI patients. In addition, because of the action of these growth factors and bioactive-cytokines, PRIMER could have beneficial effects on endometrial receptivity.

A limitation of this study is that it is not randomized. However, the KPI-score (female-age, anti-Müllerian hormone (AMH) levels, number of metaphase-II oocytes, fertilization rate, and morphological quality of embryos-transferred) and cycles characteristics including male-age, FSH total dose, mean number of oocytes retrieved, mean number of embryos-transferred, and etiology of infertility found in the study population were similar between the Study and Control groups. These findings confirm that the groups were adequately matched, minimizing bias in the study outcomes.

In conclusion, our results showed, for the first time, evidence that this therapeutic protocol (PRIMER) could be used as a feasible treatment based on biological rationale for patients with RIF, since these patients with about four previous ETs achieved ongoing pregnancy rates similar to patients who performed their first IVF/ICSI attempt cycle. The use of a rigorous match based on the KPI-score provides relevance to the outcomes found. In order to confirm the results found in this study, randomized controlled trials in a large population should be carried out.
